# Prescription drug diversion, misuse, and abuse among people living with HIV: a scoping review protocol

**DOI:** 10.1186/s13643-020-1273-4

**Published:** 2020-02-12

**Authors:** Buyisile Chibi, Neusa Fernanda Torres, Tivani P. Mashamba-Thompson

**Affiliations:** 1 Discipline of Public Health Medicine, School of Nursing and Public Health, University of KwaZulu-Natal, Durban, 4001 South Africa; 2 HIV/AIDS, STIs and TB Research Programme, Human Sciences Research Council, Durban, South Africa; 3 Centre for the AIDS Programme of Research in South Africa, Doris Duke Medical Research Institute, 2nd Floor, 719 Umbilo Road, Durban, 4041 South Africa; 4 ISCISA—Higher Institute for Health Sciences, Maputo, Mozambique; 5 Department of Public Health, Faculty of Health Sciences, University of Limpopo, Mankweng, South Africa

**Keywords:** People living with HIV, Prescription, Drug, Diversion, Misuse, Abuse, Adherence

## Abstract

**Background:**

Prescription drugs are controlled medicines due to their potential risks of being diverted, misused, and abused. Since the introduction of antiretroviral (ARVs) drugs, HIV is currently regarded as a chronic condition. However, prescription drug diversion, misuse, and abuse might serve as one of the critical barriers for achieving optimal medication adherence among people living with HIV, thereby negatively impacting the HIV care mandate. The primary aim of this scoping review is to gather evidence on the prevalence, practices, risk factors, and motives associated with prescription drug diversion, misuse, and abuse, as well as the evidence on the association between prescription drug diversion, misuse, and abuse with antiretroviral treatment (ART) adherence.

**Methods:**

This review will be guided by Arksey and O’Malley’s framework as well as recommendations by Levac et al. (Implement Sci 5:69, 2010). We will search the following databases for relevant literature meeting our eligibility criteria: PubMed, Google Scholar, EBSCOhost (Academic Search Complete, MEDLINE, and Newspaper Source), World Health Organization, Science Direct, and Open Access Theses and Dissertations. Studies published within the period of January 1996 to June 2019 are eligible. The included studies should report evidence on the prevalence, practices, risk factors, motives, or association between ART adherence and prescription drug diversion, misuse, and abuse. Thematic analysis will be applied to summarize the review findings.

**Discussion:**

We anticipate finding a considerable number of research studies on prescription drug diversion, misuse, and abuse among people living with HIV. Our synthesis of this evidence base is intended to serve as guidance for future research studies. The study findings will be disseminated through the traditional academic platforms, such as peer-reviewed publications and presentations at relevant local and international conferences, symposiums, and seminars.

**Systematic review registration:**

PROSPERO CRD42017074076

## Background

### Definitions

This review will focus on prescription drugs, defined as controlled medicines that legally would require a medical prescription from a health care provider in order to be dispensed due to their potential risks of being diverted, misused, and abused [1]. Adapted from Inciardi et al. (2016), our definition of prescription drug diversion is the illegal way of acquiring or distributing controlled medicinal drugs for any use [2]. We define prescription drug misuse according to Casati et al*.* (2012) as the inappropriate use of or not adhering to the information leaflet of a medicine [3]. We define prescription drug abuse according to Akerele et al. (2017) as improper use of a medicine in amounts or with methods that might be harmful [4]. In this study, we use the terms “drug,” “medication,” “medicine,” and “pharmaceuticals” interchangeably, and prescription drug abuse will refer to medication abuse, medicine abuse, and pharmaceutics abuse.

### Global overview of prevalence of prescription drug diversion, misuse, and abuse

Globally, prescription drug diversion, misuse, and abuse is an escalating public health problem [1]. Its impact is associated with a high likelihood of poor health care outcomes, increased incarceration cases, and increased mortality rates [1]. Drug classes commonly diverted, misused, and abused worldwide are sedatives, analgesics, and stimulants [5, 6]. The United Nations Office on Drugs and Crime estimate that, in 2014, 35.7 million people globally used stimulants (such as amphetamine and prescription stimulants), 33.12 million used analgesic (such as opiates and prescription opioids), and 207,400 people died from causes related to drug use globally [7].

### Global overview of human immunodeficiency virus (HIV)

Despite extensive efforts to eradicate HIV, the negative impact of HIV is still a public health concern [8, 9]. Globally, about 37.8 million people were diagnosed with HIV in 2018 [10]. In 2017, about 21.7 million people living with HIV (PLWH) were initiated on antiretroviral treatment (ART) while only 17.5 million had viral suppression [11, 12]. However, antiretroviral drug diversion is an emerging phenomenon (with trends similar to sedatives, analgesics, and stimulants) that is likely to negatively impact ART adherence, therefore increasing chances of treatment failure [13, 14] given that the efficacy of ART is highly dependent on optimal adherence to the treatment [15, 16].

### Guidelines and policies

The American Society of Health-System Pharmacist guidelines were put in place to prevent the diversion of controlled substances [17]. These guidelines were developed and implemented because drug diversion compromises the safety of patients, endangers the diverter, and also leads to substantial liability risk to the affected organization [17]. Nevertheless, most countries have additional policies and guidelines. For example, in South Africa, the Medicines Control Council regulates the manufacture, distribution, sale, and marketing of medicines, and the National Drug Policy (NDP) was developed and has been implemented since 1996 to prevent drug diversion, misuse, and abuse [18, 19]. However, a study evaluating the impact of the NDP found that only 30% of all participating individuals were adherent to their medication, and 29% used medication not prescribed by a health care worker [17]. These findings emphasize the need to further investigate prescription drug diversion, misuse, and abuse.

### Aim of the study

The primary aim of this review is to gather evidence on the prevalence (the extent of the problem), practices (what do people do?), risk factors (such as demographics, socio-economic status, health care, and other risk factors for prescription drug diversion, misuse, and abuse), motives (what are the reasons?), and the association with ART adherence of prescription drug diversion, misuse, and abuse.

The review findings will present a snapshot of the global status of prescription drug diversion, misuse, and abuse among PLWH, thereby enabling researchers to identify research gaps. A recently published scoping review study revealed limited research investigating risk factors for prescription drug diversion in low-middle-income countries among PLWH [20]. Our review findings will inform policy-makers, authorities from the department of health and law enforcement, and the general public about the extent of the problem, practices, risk factors, and motives associated with prescription drug diversion, misuse, and abuse to guide the design and development of tailored prevention measures.

## Methodology

### Scoping review

Peer-reviewed literature and grey literature of primary studies with various study designs addressing prescription drug diversion, misuse, and abuse will be reviewed in this scoping review. The scoping review method was chosen since it gathers evidence and allows the mapping of all existing literature and gaps [21]. The proposed scoping review will use Arksey and O’Malley’s framework [22], which is comprised of six stages: (1) identifying the research question; (2) identifying relevant studies; (3) study selection; (2) charting the data; (5) collating, summarizing, and reporting the results; and (vi) consultation exercise (optional). The subsequent sub-sections will describe the framework stages in detail [22]. Furthermore, Levac et al. (2010) recommendations for quality assessment of eligible studies will be performed [23]. The Preferred Reporting Items for Systematic Review and Meta-Analysis Extension for Scoping Reviews (PRISMA-ScR) checklist will be used as a guide for planning and documentation of the review methods [24].

### Framework stage 1: identifying the research question

The proposed review will apply the PCC (population, concept, context) mnemonic recommended for scoping reviews to define the eligibility of the research question (Table 1) [25].

**Table 1:** PCC (population, concept, context) framework for determining the eligibility of research questions

Criteria	Determinants
Population	People living with HIV age (18 years and above)
Concept	Prescription drug diversion, misuse and abuse
Context	Prevalence, practices, motives, risk factors, and ART adherence

The main research question for this study is as follows: What evidence exists on prescription drug diversion, misuse, and abuse among people living with HIV (PLWH)?

Research sub-questions are as follows:

1. What is the prevalence of prescription drug diversion, misuse, and abuse?
2. What practices of prescription drug diversion, misuse, and abuse exist?
3. What are the motives of prescription drug diversion, misuse, and abuse?
4. What risk factors impact vulnerability to prescription drug diversion, misuse, and abuse?
5. Is there any association between prescription drug diversion, misuse, and abuse with ART adherence?

### Framework stage 2: identifying relevant studies

This framework consists of three steps. The first step is selection and searching through electronic databases and data sources in order to identify both published and unpublished research studies relevant to the research question. We plan to select and search through PubMed, Google Scholar, EBSCOhost (Academic Search Complete, MEDLINE and Newspaper Source), World Health Organization, Science Direct, and Open Access Theses and Dissertations. The second step involves the use of keywords such as people living with HIV, prescription, drug, diversion, misuse, abuse, and adherence. These keywords will be used to search through the selected databases and data sources for primary studies either from peer-reviewed journals or from grey literature addressing the research question. The third step involves a further search of the reference lists of all identified articles and reports for additional eligible studies.

### Framework stage 3: study selection

Inclusion and exclusion criteria will be used to ensure consistent exclusion of studies that do not address the research question. The eligibility criteria will be established to ensure that the selected and included studies cover relevant information required to answer the research question of prescription drug diversion, misuse, and abuse among PLWH.

### Eligibility criteria

**Fig. 1:**
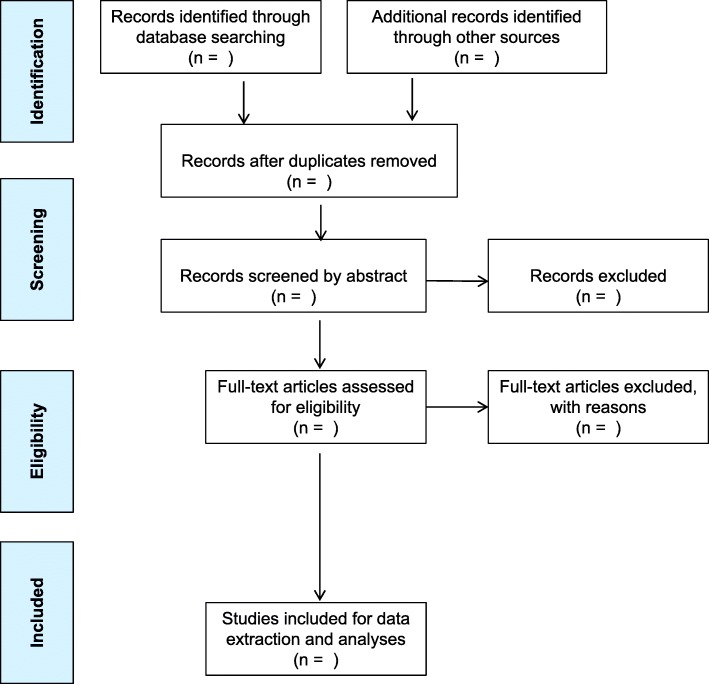
Study selection flowchart

Studies will either be selected or rejected according to the eligibility criteria detailed in Table 2. The search will be restricted to studies published from January 1996, which was the year marking the onset of the highly active antiretroviral therapy era, therefore allowing us to better track ARVs usage, other prescription drugs, and other questions of interest among PLWH [26]. The most common drug classes with great potential of diversion, misuse, and abuse are sedatives, analgesics, stimulants, Z-drugs, anesthetic drugs, and antiretroviral drugs [5, 6]. PLWH often present with chronic illnesses (such as cancer, neurological complications, and tuberculosis) that may require prescription drugs in addition to ART. Chronic pain has been commonly diagnosed among PLWH and often treated with opioids [16]. We will use Endnote to manage records, keep track of articles, and make requests for inter-library loans. Based on a pilot search demonstrating feasibility, one reviewer will search for articles and reports from the selected databases by using the following keywords: PLWH, prescription, drug, medication, diversion, misuse, abuse, recreational, non-medical use, illicit, selling, sharing, giving away, trading, stealing, missing/losing, doctor shopping, pharmacy shopping, prevalence, practice, risk factor, motive, and adherence. The Boolean operators, i.e. AND and OR terms will be used to combine keywords into phrases during the title search. Eligible titles will be exported to EndNote ×8 (Thomson Reuters, New York, USA) reference management software and later shared among reviewers. Two reviewers will conduct a comprehensive screening of the abstracts and full articles guided by the eligibility criteria in order to make conclusions on which studies to include for the scoping review. Discrepancies between reviewers’ responses at abstract screening will be resolved by discussion among the project team members, while discrepancies at the full article screening stage will be resolved by a third reviewer. In case where full-text articles are not freely available, a request to retrieve the full-text will be sent to the University of KwaZulu-Natal and Human Sciences Research Council library service. Table 3 illustrates how the electronic data search will be recorded. The number of citations at each stage of study selection will be reported in a PRISMA flowchart (see Fig. 1) [27].

**Table 2:** Inclusion and exclusion criteria for determining eligible studies

	Inclusion criteria	Exclusion criteria
Language	English	None English language studies
Population	People living with HIV (PLWH)	Studies which do not focus on PLWH or do not include PLWH
Period	Studies published from 1 January 1996 to 30 June 2019	Studies published before January 1996
Type of studies	Primary and secondary studies	Review articles (including systematic review and meta-analysis) However, reference list from review articles will be searched for further eligible studies
Studies designs	Quantitative Qualitative Mixed methods Randomized controlled trial Cohort study (prospective observational study) Case-control study Cross-sectional study Case reports and series Ideas, editorials, opinions Crossover design	Animal research studies Test-tube lab research
Study focus	Studies that report on either of the following: • Prescription drug diversion • Prescription drug misuse • Prescription drug abuse • Recreational/non-medical use/misuse/abuse/illicit/selling/trading/sharing/giving away/stealing/losing, and doctor shopping of prescribed drugs • Prevalence of prescription drug diversion, misuse or abuse • Practices of prescription drug diversion, misuse or abuse • Motives (other terms such as reasons, cause, purpose, intention, motivation, drive, and aim maybe used) of prescription drug diversion, misuse, or abuse • Risk factors of prescription drug diversion, misuse, or abuse • ART adherence	Studies that do not focus or report on the following: • Prescription drug diversion, misuse or abuse • Prevalence, practices, motives, risk factors and ART adherence associated with prescription drug diversion, misuse or abuse
Drug classes	Studies that report on diversion, misuse, and abuse of the below drug classes: • Sedatives—including barbiturates, benzodiazepines, and sleep medications • Analgesics—including opioids heroin, morphine, hydromorphone, meperidine, oxycodone, hydrocodone, oxycodone, buprenorphine, methadone, fentanyl, and codeine • Stimulants—including cocaine, methamphetamine, amphetamine, and methylphenidate • Z-drugs (nonbenzodiazepines)—including imdazopyridines, cycloprrolones, pyrazolopyridines, and sleep medication • Anesthetic drugs • Antiretroviral drugs • Attention deficit hyperactivity disorder (ADHD) medication • Cannabinoids	Studies that report on diversion, misuse, and abuse of the below drug classes will be excluded: • Ecstasy drug—since it has no accepted medical indication

**Table 3:** Electronic search record using keywords (pilot study)

Date and time	Keyword search	Search engine used	Number of publication retrieved	Number of duplicates
16 August 2017	Drug diversion (filtered by publication year 01/01/1996 to 31/07/2017)	PubMed	1852	0
16 August 2017	Prescription drug diversion (filtered by publication year 01/01/1996 to 31/07/2017)	PubMed	556	0
16 August 2017	Prescription drug misuse (filtered by publication year 01/01/1996 to 31/07/2017)	PubMed	9459	0
16 August 2017	Prescription drug abuse (filtered by publication year 01/01/1996 to 31/07/2017)	PubMed	11193	0

### Framework stage 4: charting the data

A charting form (see Table 4) will be utilized to record characteristics and relevant key information of the included studies addressing the review question. The charting form will frequently be refined and tested to ensure that important information is extracted and to check the appropriateness of the identified databases and keywords. The extracted results from the charting form will be presented as tables.

**Table 4:** Data charting form

Author and date	
Study title	
Journal full reference	
Aims or research question	
Participants characteristics	
Recruitment methods	
Sampling method	
Study design	
Data collection (what data collection methods were used?)	
Data analysis (how was the data analyzed?)	
Exposures/interventions	
Outcome	
Most relevant finding	
Conclusions	
Comments	

### Framework stage 5: collating, summarizing, and reporting the results

This study will present a narrative account of findings from eligible studies through a thematic analysis of the extracted research data. The themes will be structured around the following anticipated outcomes: prevalence, practices, risk factors, motives, and ART adherence. This study will also consider emerging themes. The researchers will consider the use of a google form during data extraction to assist with organizing data. NVIVO software version 10 [28] will be utilized during thematic analysis. Thematic analysis will be used for identifying, analyzing, and reporting patterns (themes) within data.

The researchers will follow the below steps:

- ❖ Familiarize themselves with data (through reading) of all eligible studies, considering the research question and anticipated outcomes
- ❖ Use a google form to capture extracted data addressing the research question
- ❖ Generate codes of the extracted data to describe the themes or patterns
- ❖ Categorize codes into major themes
- ❖ Display the categorized extracted data and review themes
- ❖ Identify patterns and define themes and sub-themes
- ❖ Summarize data

The researchers will cross-examine the themes in relation to the asked research question, therefore scrutinizing the meaning of the review results as well as identifying research gaps warranting for future research.

### Framework stage 6: consultation exercise

This review will not include the consultation exercise stage.

### Quality assessment

The quality of the searched studies will be assessed through the mixed method appraisal tool (MMAT)-Version 2011 [29]. The MMAT allows for simultaneous assessment and illustration of the methodological quality of mixed, qualitative, and quantitative (including randomized controlled, nonrandomized, and descriptive) methodological domains [29]. This tool will be used to examine the relevance of the study aim, methodology, study design, sample framework, data collection, data analysis, presentation of findings, authors’ discussions and conclusions, and the overall quality of each study.

## Discussion

This scoping review aims to gather evidence on the prevalence, practices, motives, and risk factors associated with prescription drug diversion, misuse, and abuse, as well as the association between prescription drug diversion, misuse, and abuse with ART adherence among people living with HIV. Prescription drug diversion, misuse, and abuse is an emerging phenomenon in low-middle-income countries, yet this area of research had received little attention in scientific research even though the media continuously reports new cases. This review will allow authors to further explore what is known and unknown about prescription drug diversion, misuse, and abuse.

Limitations in this scoping review will arise due to the focus on mapping the breadth of studies instead of the depth of information, in order to identify research gaps. However, the chosen methodology is appropriate in addressing the review questions. As well, we limited the included studies to those of prescription drugs; studies that report drugs such as ecstasy will be excluded since their medical use is not scientifically established and not prescriptible by a health care worker.

The review findings may be of interest to policy-makers involved in projects for eradicating barriers to achieving 100% ART adherence and to law enforcement officers aimed at reducing and possibly stopping drug diversion. Furthermore, the review findings will be of interest to researchers by highlighting research gaps that may need further investigation.

## Data Availability

All data generated or analyzed during this study will be included in the published scoping review article as supplementary material.

## Abbreviations

ART: Antiretroviral therapy
ARVs: Antiretrovirals
HIV: human immunodeficiency virus
MMAT: Mixed methods appraisal tool
NDP: National Drug Policy
PCC: Population, concept, context
PLWH: People living with HIV
PRISMA-ScR: Preferred Reporting Items for Systematic Review and Meta-Analysis Extension for Scoping Reviews
